# Herpes simplex virus type-1(HSV-1) oncolytic and highly fusogenic mutants carrying the NV1020 genomic deletion effectively inhibit primary and metastatic tumors in mice

**DOI:** 10.1186/1743-422X-5-68

**Published:** 2008-06-02

**Authors:** Anna Israyelyan, Vladimir N Chouljenko, Abolghasem Baghian, Andrew T David, Michael T Kearney, Konstantin G Kousoulas

**Affiliations:** 1 Division of Biotechnology and Molecular Medicine and Department of Pathobiological Sciences, School of Veterinary Medicine, Louisiana State University, Baton Rouge, LA 70803, USA; 2 Department of Pathobiological Sciences, School of Veterinary Medicine, Louisiana State University, Baton Rouge, LA 70803, USA

## Abstract

**Background:**

The NV1020 oncolytic herpes simplex virus type-1 has shown significant promise for the treatment of many different types of tumors in experimental animal models and human trials. Previously, we described the construction and use of the NV1020-like virus OncSyn to treat human breast tumors implanted in nude mice. The syncytial mutation gKsyn1 (Ala-to-Val at position 40) was introduced into the OncSyn viral genome cloned into a bacterial artificial chromosome using double-red mutagenesis in *E. coli *to produce the OncdSyn virus carrying syncytial mutations in both gB(syn3) and gK(syn1).

**Results:**

The OncdSyn virus caused extensive virus-induced cell fusion in cell culture. The oncolytic potential of the OncSyn and OncdSyn viruses was tested in the highly metastatic syngeneic mouse model system, which utilizes 4T1 murine mammary cancer cells implanted within the interscapular region of Balb/c mice. Mice were given three consecutive intratumor injections of OncSyn, OncdSyn, or phosphate buffered saline four days apart. Both OncSyn and OncdSyn virus injections resulted in significant reduction of tumor sizes (p < 0.05) compared to control tumors. Virus treated mice but not controls showed a marked reduction of metastatic foci in lungs and internal organs. Mouse weights were not significantly impacted by any treatment during the course of the entire study (p = 0.296).

**Conclusion:**

These results show that the attenuated, but highly fusogenic OncSyn and OncdSyn viruses can effectively reduce primary and metastatic breast tumors in immuncompetent mice. The available bac-cloned OncSyn and OncdSyn viral genomes can be rapidly modified to express a number of different anti-tumor and immunomodulatory genes that can further enhance their anti-tumor potency.

## Background

Recent advances in molecular virology have enabled investigators to construct viruses that selectively destroy cancer cells (oncolytic virotherapy). Genetically engineered viruses belonging to different viral families have been evaluated for their potential as therapeutic agents in the treatment of malignant tumors [1-4]. Efficient replication, cell lysis and spread of HSV, and their natural broad host range make them attractive candidates as oncolytic viral agents [5-7]. Furthermore, the recent availability of cloned HSV genomes into bacterial artificial chromosome vectors greatly facilitates the rapid construction of new recombinant viruses carrying multiple transgenes of interest [8-10]. Tumor treatment with oncolytic HSV has been shown to induce anti-tumor immune responses [11-15]. Although the majority of people are seropositive for HSV-1, oncolytic virotherapy with HSV is not limited by pre-existing anti-HSV immunity [16,17], and in at least one example, preexisting immunity to HSV-1 enhanced anti-tumor immune responses [18].

Recently, the NV1020 oncolytic herpes simplex virus type-1(HSV-1) was shown to have significant promise for the treatment of many different types of tumors in preclinical studies in experimental animals as well as in human clinical trials [17,19-22]. The main advantage of this virus over other HSV oncolytic viruses is that it expresses one of the two original γ_1_34.5 genes allowing the virus to replicate more efficiently, while safety is not compromised [23-26]. The γ_1_34.5 gene is a major neurovirulence gene and an inhibitor of cellular apoptosis. Deletion of this gene drastically attenuates the virus and restricts viral growth to cancer cells because of their lack of intact apoptotic mechanisms [27,28]. Preclinical studies in mice as well as phase I/II human trials have revealed that oncolytic HSV-1 viruses having both γ_1_34.5 genes deleted did not spread efficiently within tumors [29]. In contrast, deletion of one of the two γ_1_34.5 genes drastically attenuated the virus, while allowing improved virus replication and spread in tumor cells [23-25]. The NV1020 was originally constructed for vaccine purposes and it contains HSV-2 viral sequences coding for glycoproteins gD, gG, gI and gE to facilitate production of anti-HSV-2 immune responses [24].

HSV can be transmitted from cell-to-cell by causing limited amounts of virus-induced cell fusion, thus avoiding the extracellular environment. Specific mutations within viral glycoproteins are known to greatly enhance virus-induced cell fusion. Specifically, syncytial mutations that cause extensive virus-induced cell fusion can arise in at least two of the glycoprotein genes: the UL27 gene, encoding glycoprotein B (gB) [30-32], and the UL53 gene, coding for glycoprotein K (gK) [33,34]. Work in our laboratory has shown that gK functions as a heterodimer with the UL20 viral protein and the UL20/gK heterodimer is necessary for virus-induced cell fusion [35,36].

The HSV-1 oncolytic virus Onc was constructed based on the NV1020 genomic arrangement with the exception that there are no genomic re-arrangements and no HSV-2 genes inserted within the viral genome. Recently, we reported that the OncSyn virus carrying a syncytial mutation in gB, enabling the virus to spread among cells by virus-induced cell fusion, replicated efficiently in breast cancer cells *in vitro *and drastically reduced tumor volumes *in vivo *[37]. In this study we constructed and tested the OncdSyn virus, which in addition to the gBsyn3 mutation also carried the gKsyn1 mutation known to enable the virus to fuse even difficult to fuse cells [38]. Intra-tumor injections of either virus effectively reduced tumor volumes as well as inhibited tumor metastases to internal organs.

## Results

### Construction and characterization of the Oncolytic HSV-1 mutant virus OncdSyn

**Figure 1:**
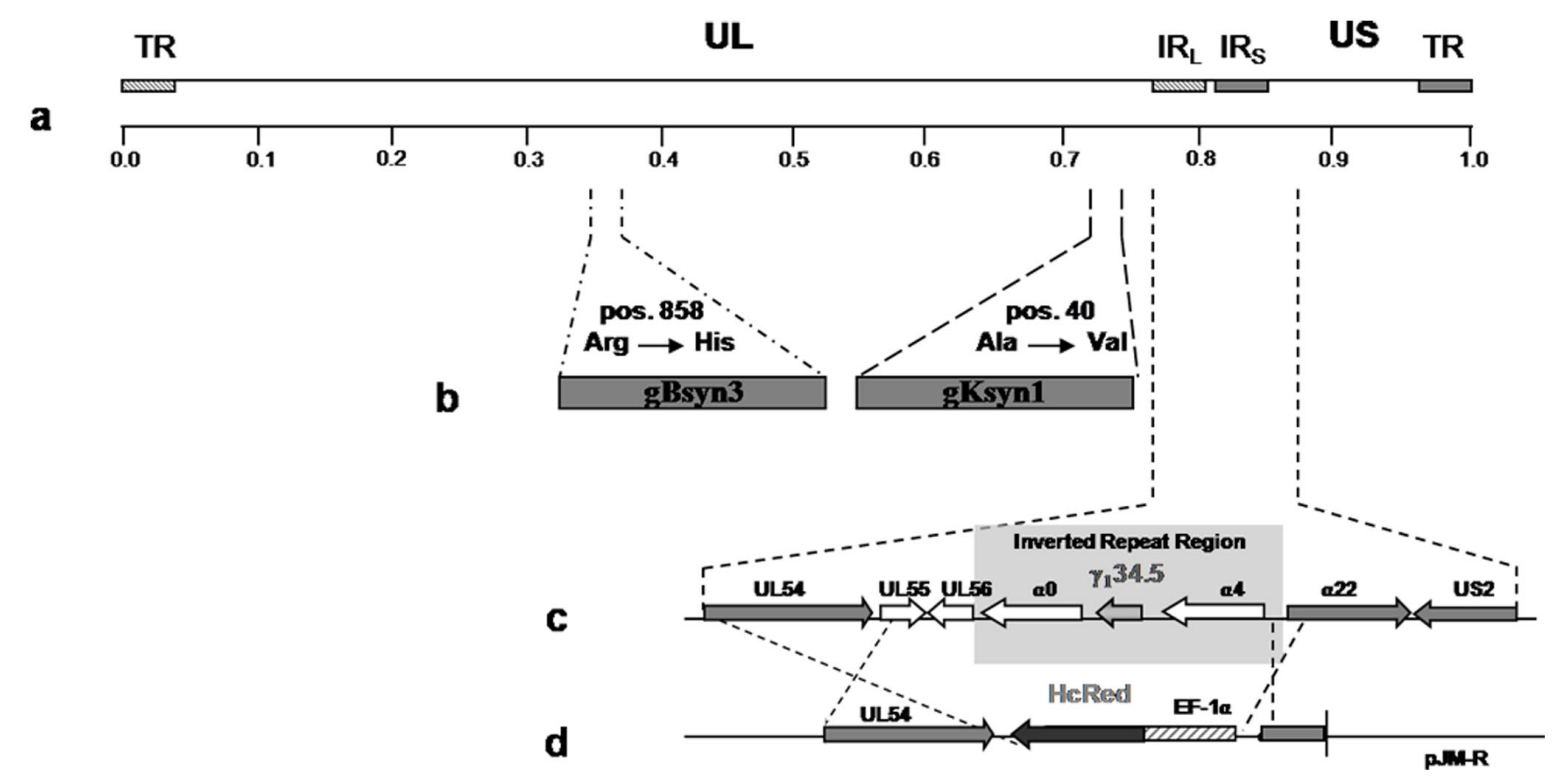
**Schematic representation of the genomic structures of the oncolytic recombinant viruses OncSyn and OncdSyn**. (a) Representation of the prototypic arrangement of the HSV-1 genome with the unique long (UL) and unique short (US) regions flanked by the terminal repeat (TR) and internal repeat (IR) regions. (b) Approximate locations of the gB and gK genes. (c) An expansion of the inverted repeat region showing the approximate locations of UL54, UL55, UL56, α 0, γ_1_34.5, α 4, α 22 and US2 genes. (d) Schematic of the DNA fragment cloned into plasmid pJM-R, which was used for insertion of the HcRed gene cassette into the viral genome in place of the NV1020 genomic deletion as described in Materials and Methods.

Previously, we described the construction and use of the NV1020-like virus OncSyn to treat human breast cancer utilizing a nude mouse xenograft model [37]. To further increase the ability of the OncSyn virus to cause virus-induced cell fusion, the syncytial mutation gKsyn1 (Ala-to-Val at position 40) known to cause virus-induced cell fusion of even hard to fuse cells [38] was introduced into the OncSyn viral genome cloned into a bacterial artificial chromosome (bac) using the markerless double-red mutagenesis method [39]. The resultant OncdSyn virus carried syncytial mutations in both gB (syn3) and gK (syn1) (Fig. 1). As we reported previously for the OncSyn virus, the bac-cloned OncdSyn viral genome was subjected to PCR-diagnostic analysis and direct sequencing of specific genomic loci to confirm the presence of the syn3 and syn1 mutations and the previously engineered deletion/insertion at the γ_1 _34.5 locus (not shown, Materials and Methods).

### Phenotypic characteristics of the OncSyn and OncdSyn viruses on Vero and 4T1 cells

**Figure 2:**
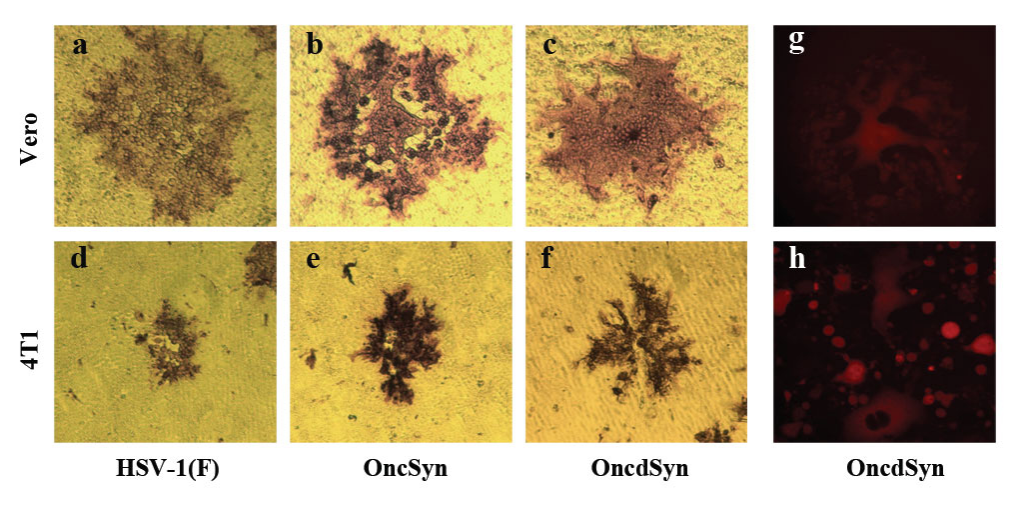
**Plaque morphology of the HSV-1 (F), OncSyn and OncdSyn viruses**. Nearly confluent Vero (a-c) and 4T1 (d-f) cell monolayers were infected with wild-type HSV-1(F) (a, d), OncSyn (b, e) and OncdSyn (c, f) viruses. Individual viral plaques were visualized 48 hr post infection by immunohistochemistry and photographed with a phase contrast microscope. Vero (g) and 4T1 (h) cells were infected with OncdSyn virus. Viral plaques were photographed 48 hr postinfection with a fluorescent microscope.

The plaque morphology of the HSV-1(F), OncSyn and OncdSyn viruses was examined on Vero cells and 4T1 cancer cells (Balb/c spontaneous mammary adenocarcinoma-derived) [40] as described in Materials and Methods (Fig. 2). Plaque morphologies were visualized on Vero and 4T1cells at 48 hours post infection (hpi) by immunohistochemistry using a polyclonal anti-HSV-1 antibody (Fig. 2a–f). Mouse cells are known to be resistant to HSV-1 infection [41,42]. Consequently, viral plaques generated by all three viruses tested were substantially smaller on 4T1 mouse cancer cells (Fig. 2d, e, f) in comparison to Vero cells (Fig. 2a, b, c). Specifically, the HSV-1(F) wild-type virus, which does not cause extensive virus-induced cell fusion, produced viral plaques on 4T1 cells that were approximately 10-fold smaller than those produced on Vero cells (Fig. 2d and 2a). In contrast, the OncSyn and OncdSyn viruses produced syncytial plaques on both cell lines tested (Fig. 2b, c, e, f); however, both the OncSyn and OncdSyn viral plaques on 4T1 cells were larger than those produced by the HSV-1(F) wild-type virus (Fig. 2e and 2f compared to d). The OncdSyn virus appeared to cause more pronounced virus-induced cell fusion on both Vero and 4T1 cells (Fig. 2c and 2f). In addition, the OncdSyn viral plaques emitted strong red fluorescence due to constitutive expression of the red fluorescence protein (RFP) expressed under the elongation factor 1α (EF-1α) promoter control (Fig. 2g and 2h), as it was previously reported for the OncSyn virus [37].

### Kinetics of viral replication on Vero and 4T1 cells

**Figure 3:**
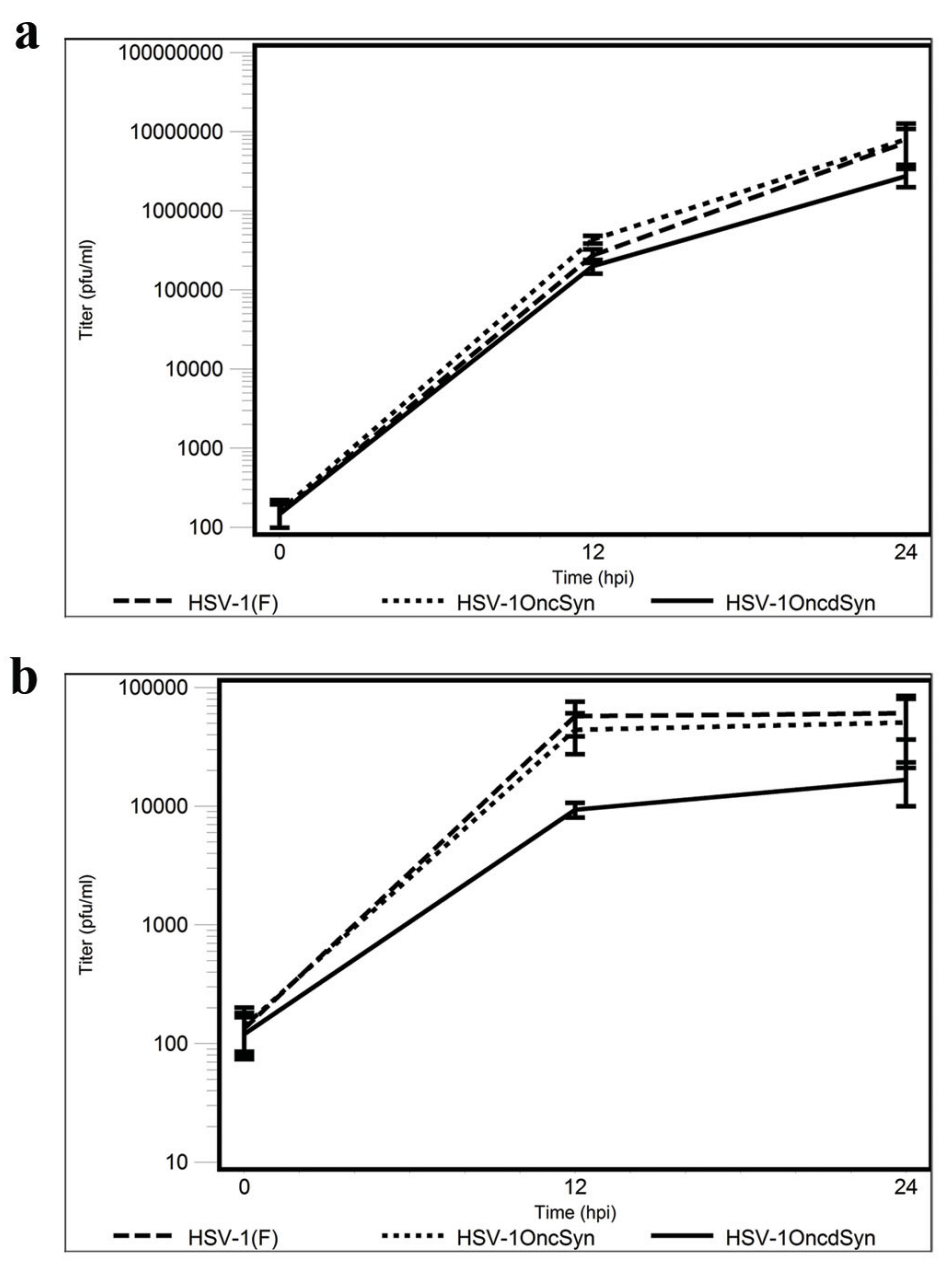
**Comparative kinetics of viral replication of wild-type HSV-1(F) and mutant viruses OncSyn and OncdSyn grown on Vero and 4T1 cells**. Near confluent monolayers of Vero (a) and 4T1 (b) cells were infected at an MOI of 2 with each virus, incubated at 37°C and the numbers of infectious virions were determined at different times post infection. Viral titers (mean pfu at each time point) are shown in logarithmic scale. The error bars represent means ± 2 standard errors.

HSV-1(F) and OncSyn viruses replicated to similar titers in Vero cells, while the OncdSyn virus consistently replicated to titers that were a half-log lower than either HSV-1(F), or OncSyn viruses. The kinetics of viral replication were substantially slower in 4T1 cells than in Vero cells, and final titers in 4T1 cells were more than two logs lower for HSV-1(F) and OncSyn, while OncdSyn viral titers were more than three logs lower on 4T1 cells than in Vero cells. In addition, OncdSyn viral titers were approximately one log lower than the HSV-1(F) and OncSyn viral titers on 4T1 cells (Fig. 3).

### Intra-tumor virotherapy

**Figure 4:**
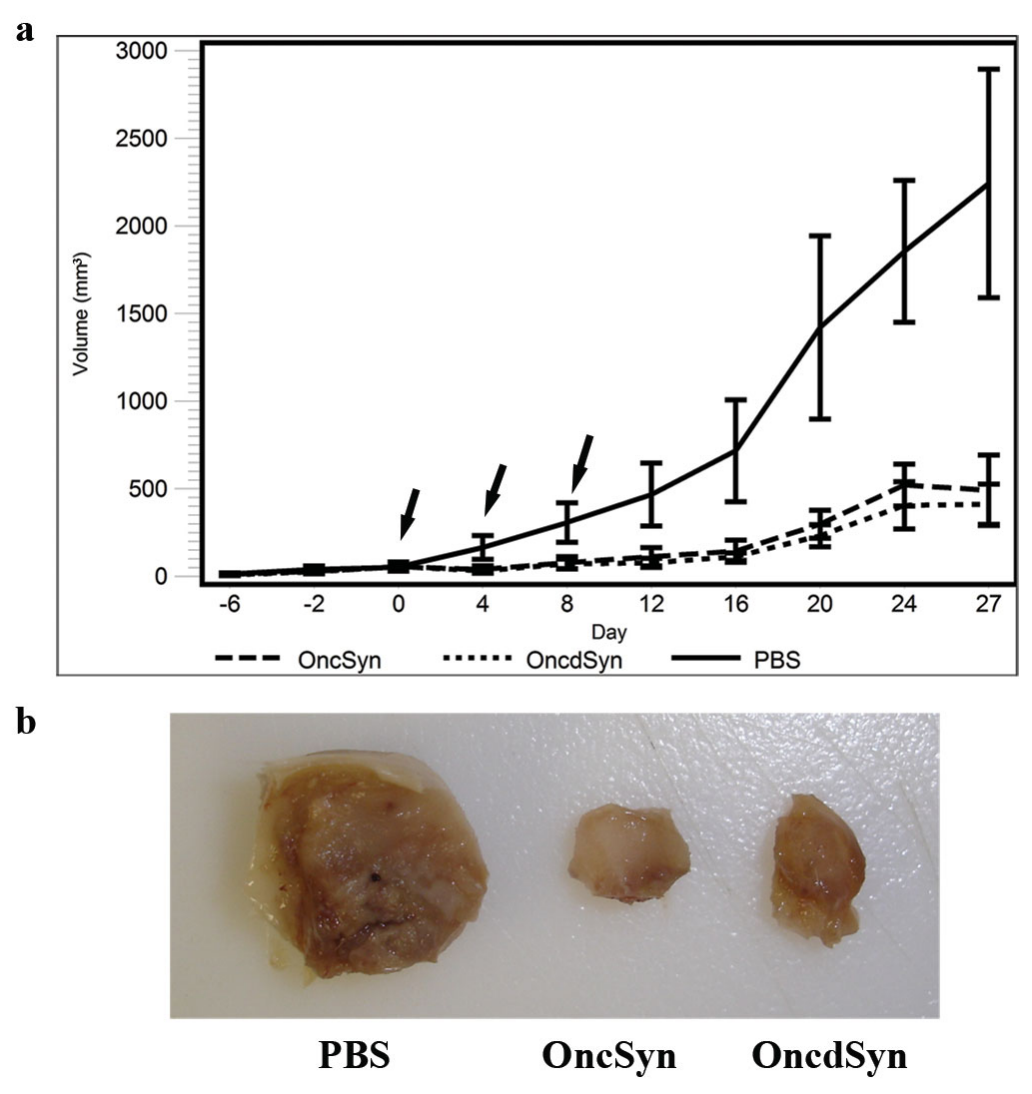
**Intra-tumor treatment with OncSyn and OncdSyn viruses**. (a) Balb/c mice were implanted subcutaneously in the interscapular area with 1 × 10^5 ^viable 4T1 cells. Tumors were measured using a digital caliper at defined time intervals prior and after treatment (x axis). Tumors were injected with either OncSyn, OncdSyn viruses, or PBS when tumors reached approximately 80–90 mm^3 ^in volume. Tumor volumes were measured prior to (negative values on the x axis) and after the injections. "0" on X axis represents the day of the first injection. The tumor volumes were determined from the formula: volume = (length × width × height)/2. Arrows indicate the days when therapy was administered. The error bars represent means ± 2 standard errors. (b) Tumors were excised at 42 days post implantation and visually examined. Panel shows representative tumors from virus and PBS treated animals.

4T1 cells were injected subcutaneously in the interscapular regions of Balb/c female mice. When the palpable tumors reached the volume of approximately 80–90 mm^3^, mice were injected with three consecutive intra-tumor injections of OncSyn and OncdSyn viruses or PBS (control) every four days as described in Materials and Methods. At the onset of viral intratumor injections, tumor sizes appeared similar in size for all three groups of mice (p > 0.05). Intratumor treatment with either OncSyn or OncdSyn virus caused a substantial reduction of tumor volumes in comparison to the PBS-treated control group of mice (p < 0.05). There was no significant difference in the reduction of tumor sizes in the two viral groups when compared to each other (p > 0.05) (Fig. 4a). Analysis of mouse weights during the course of the study did not show significant differences among the three groups, thus the efficacy of treatments was not affected by differential weight gain/loss in the groups (not shown) (p = 0.296). Representative tumors were excised immediately after mice were sacrificed. Typically, tumors treated with the PBS control injections appeared substantially larger than those treated with either the OncSyn or OncdSyn viruses (Fig. 4b).

**Figure 5:**
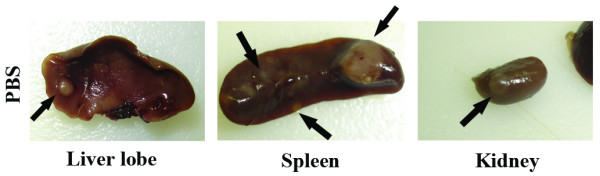
**Gross pathological examination of metastatic tumor nodules on internal organs**. Liver lobe, spleen, and kidney from a PBS-treated mouse carrying metastatic tumors (arrows) evaluated by gross pathological examination. Panel shows internal organs derived from a representative PBS-treated mouse.

The metastatic potential of the primary 4T1 tumor to internal organs after oncolytic or control therapy was assessed by gross and microscopic pathological examination of internal organs. In the first experimental protocol described above, mouse tumors were allowed to grow to approximately 80–90 mm^3 ^and mice were sacrificed at 42 days post tumor cell implantation. In this experiment, mouse lungs from all three groups of mice (PBS, OncSyn, OncdSyn) had numerous metastatic foci, which were too numerous to be accurately counted (not shown). However, tumor foci in liver and spleen were substantially reduced in OncSyn and OncdSyn-treated mice in comparison to PBS-treated control mice (Table 1, Fig. 5). Specifically, all mice in the PBS group had metastatic nodes in liver, spleen, or kidneys. Some of the mice had tumors in all three organs (Fig. 5). Importantly, there were no metastatic tumors observed in the kidneys of virus-treated mice (Table 1).

**Table 1:** Metastatic nodes in internal organs

**Experimental groups**	**No. of mice in group**	**No. of mice with metastases in internal organs**	**No. of mice with metastases in liver**	**No. of mice with metastases in spleen**	**No. of mice with metastases in kidney**
**PBS**	**9**	9	6	7	3
**OncSyn**	**7**	4	2	2	0
**OncdSyn**	**7**	3	1	2	0

Experimental animals were sacrificed on day 42 post-injection of 4T1 cells and the internal organs were removed and examined for metastases formation by gross pathological evaluation as described in Materials and Methods.

**Figure 6:**
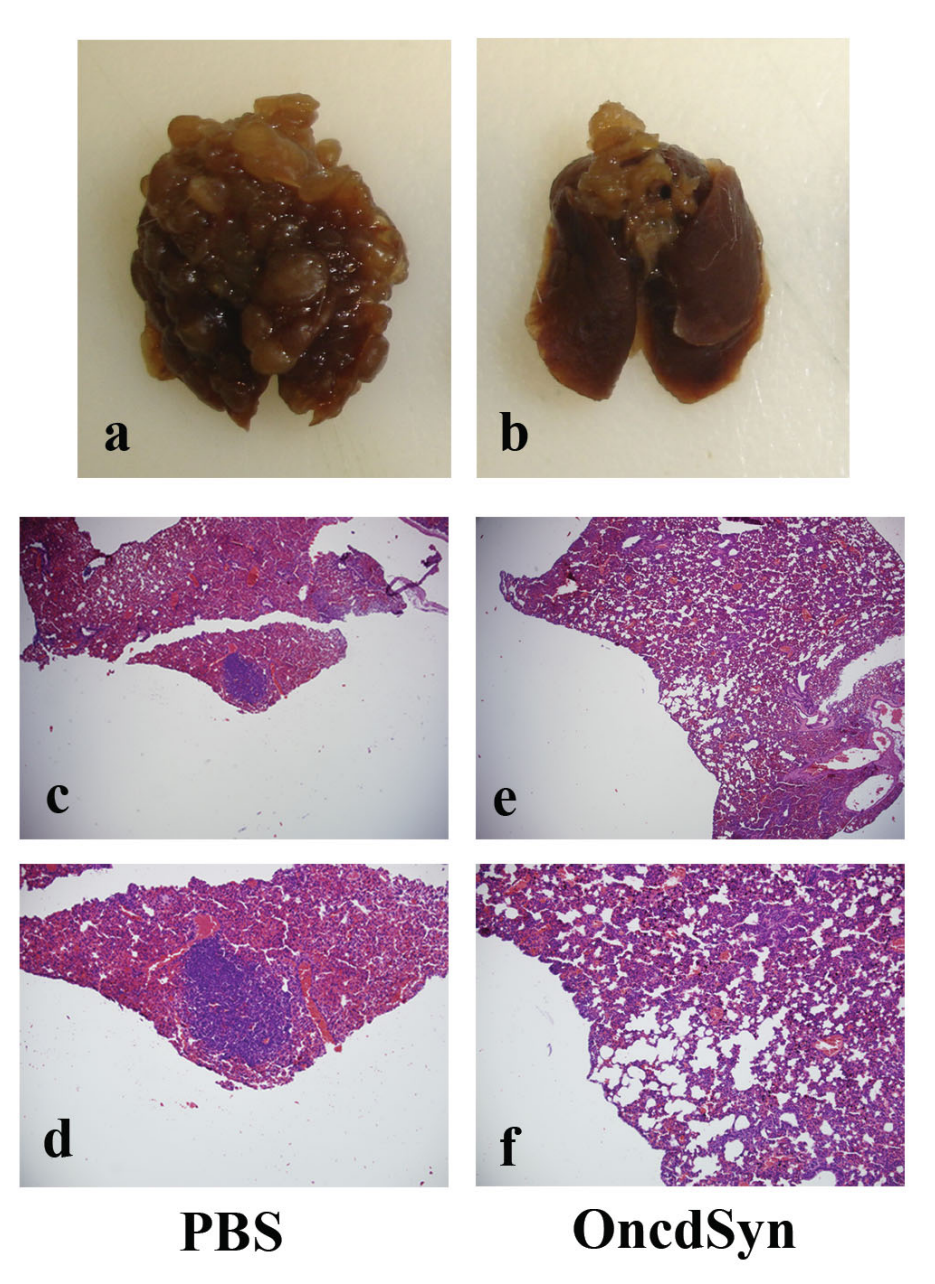
**Therapeutic effect of OncdSyn virus on lung metastases**. (a, b) Gross appearance of excised lungs of representative mice from PBS control and OncdSyn treated groups. (c-f) Lung tissues were stained with H&E and examined. Representative stained sections are shown for PBS (c, d) and OncdSyn (e, f) groups at 40× (c, e) and 100× (d, f) magnifications. Metastatic foci are represented by arrows (c, d).

To better assess the potential of oncolytic virotherapy to reduce metastatic tumors in internal organs, a second experiment was performed in a similar fashion to the previous one with the exception that in the new experiment tumors were allowed to grow to approximately 35–40 mm^3 ^in volume and mice were sacrificed at day 33 post tumor cell implantation after treatment with either OncdSyn or PBS. Lungs of OncdSyn-treated mice appeared to be practically devoid of metastatic tumors with only two mice having two nodes each. In contrast, all PBS-treated mice had multiple metastatic tumors in their lungs (Table 2, Fig. 6a and 6b). These results were confirmed by pathological examination of paraffin-embedded lung sections stained with Hematoxylin & Eosin (H&E) staining, which revealed the absence of tumors in OncdSyn samples, while PBS-treated control samples had numerous visible tumor foci (Fig. 6c–f).

**Table 2:** Metastatic nodes in lungs

**Experimental groups**	**No. of mice in group**	**No. of metastatic nodes in lungs of experimental animals**^a^							
		mouse1	mouse2	mouse3	mouse4	mouse5	mouse6	mouse7	mouse8

**PBS**	**7**	3	5	3	18	3	10	1	
**OncdSyn**	**8**	2	0	0	0	2	0	0	0

Experimental animals in PBS and OncdSyn groups were sacrificed on day 33 post-inoculation of 4T1 cells and the lungs were removed and examined for metastatic node formation as described in Materials and Methods. ^a^PBS > OncdSyn (Wilcoxon Two-Sample test, p = 0.002).

## Discussion

The oncolytic HSV-1-based virus NV1020 has shown strong promise for treatment of different tumors in animal models and human clinical trials [17,19-22]. To facilitate the construction of recombinant viruses carrying multiple transgenes of interest, we cloned the NV1020-like HSV-1 recombinant virus OncSyn into a bacterial artificial chromosome (bac) vector. The OncSyn virus specifies a syncytial mutation in gB (Arg-to-His change at aa 858) that increases its ability to spread in tumor cells via virus-induced cell fusion [37]. In this study, we introduced the syncytial mutation syn1 (Ala-to-Val change at aa 40) within the gK gene to further enhance the fusogenicity of the new virus OncdSyn. The OncdSyn virus reduced primary tumor sizes and inhibited metastases to distal organs in the 4T1 syngeneic mouse model system.

The syn1 mutation within gK has been shown to produce extensive virus-induced cell fusion in all cells tested. In comparison, the gBsyn3 mutation produced virus-induced cell fusion in most cells, but it was unable to fuse certain hard to fuse cells, such as Hep-2 cells derived from human laryngeal carcinoma [38]. Therefore, to further increase the ability of the OncSyn virus to fuse all types of cells, we generated the OncdSyn virus carrying both the gBsyn3 and gKsyn1 mutations. As expected, the OncdSyn virus caused extensive virus-induced cell fusion and fused Hep-2 cells, while the OncSyn virus did not (not shown). Furthermore, the OncdSyn virus caused more extensive fusion than OncSyn in both Vero and 4T1 cells. The OncdSyn virus appeared to produce intact syncytia that remained attached to the cell culture flasks, while the OncSyn virus-induced syncytia contained infected single cells, which detached easier than the OncdSyn-infected syncytia. This phenomenon has been previously observed for the gB and gK syncytial mutations and it is probably due, in part, to the extensive virus-induced cell fusion caused by the gK syncytial mutation, which appears to also fuse internal membranes such as nuclear membranes in addition to plasma membranes of cells (Kousoulas, unpublished). Viral titers of the OncdSyn virus were lower in Vero cells than titers of the OncSyn virus and substantially lower than titers of the OncSyn virus in 4T1 cells. Typically, HSV-1 syncytial mutants produce lower viral titers than their parental wild-type viruses, most likely because of their direct effect on cellular membranes. In this regard, the increased ability of the OncdSyn virus to cause extensive virus-induced cell fusion is probably responsible for the observed decrease in viral titers in comparison to the OncSyn virus.

Defects of viral replication and spread in mouse cancer cells have been described in the literature for oncolytic herpesviruses [11,14]. HSV-1 does not replicate efficiently in mouse cell lines [41,42] most likely because it cannot as efficiently utilize the mouse nectin-1 receptor, which is approximately 5% different in its amino acid sequence to the human nectin-1 receptor [43]. Nectin-1 is also known to facilitate virus-induced cell fusion and virus-spread [44]. Consequently, both OncSyn and OncdSyn viruses replicated much less efficiently in 4T1 cells than in Vero cells. In this regard, the limited replication and spread of these viruses in 4T1 cells would be expected to adversely affect their oncolytic ability in 4T1-derived tumors *in vivo*. Previously, we reported that the OncSyn virus effectively reduced primary human breast cancer tumors in nude mice [37]. The disadvantage of the MDA-MB-435S human breast tumors is that these tumors would be rapidly eliminated if they were implanted in immunocompetent mice. Therefore, we chose the 4T1/Balb/c mouse model system for additional testing of both the previously constructed OncSyn virus as well as the newly constructed OncdSyn virus. Both OncSyn and OncdSyn viruses substantially reduced the growth of 4T1 tumors compared to the PBS controls, despite the fact that these viruses did not efficiently replicate in 4T1 cells in cell culture. Apparently, viral replication and infectious virus production in cell cultures did not correlate with the oncolytic efficacy of these viruses, because the OncdSyn virus reduced tumor volumes equally-well with the OncSyn virus, despite the fact that OncdSyn replicated approximately less than half a log than the OncSyn virus in 4T1 cells. Therefore, the relative increased ability of the OncdSyn virus to destroy tumors *in vivo *must be attributed to its enhanced fusogenicity.

Multiple murine tumor models have been used as preclinical settings for therapeutic purposes. The 4T1 mammary carcinoma model has several distinct advantages to be used as such model. It is regarded as a highly physiological, clinically-relevant mouse model that closely resembles stage IV human breast cancer in its properties [40]. 4T1 cells are considered to be very weakly immunogenic (relative antigenic strength is less than 0.01 with 9.9 being the most immunogenic) [45,46], and they spontaneously metastasize to distal parts of the body [40,47]. Metastatic tumor foci in liver and spleen were substantially reduced in OncSyn and OncdSyn-treated mice in comparison to PBS-treated control mice. Reduction of metastatic foci in internal organs (lung, spleen, kidney and liver) was dependent on the size of the original 4T1 tumor, as well as the time of necropsy post implantation of tumor cells. Specifically, there was drastic reduction in tumor foci detected in lungs when the initial tumor size treated with the virus was approximately 35–40 mm^3 ^and necropsies were performed at 33 days after tumor implantation. Furthermore, lungs appeared to have the same number of metastatic foci with PBS-treated controls when the initial treated tumors where 80–90 mm^3 ^and necropsies were performed at day 42 after tumor implantation. This metastatic pattern revealed that lungs were the primary metastatic site of the subcutaneous implanted 4T1 cells. Regardless of the size of the initial tumor treated and the time of necropsies post tumor implantation, it was evident that OncSyn and OncdSyn viruses appeared to efficiently reduce the growth of the primary tumor as well as substantially inhibit or eliminate formation of metastatic foci.

It is highly likely that reduction of the primary tumor after oncolytic virotherapy with the OncSyn and OncdSyn viruses is responsible for the observed reduction in the formation of secondary tumor foci, since treatment of the smaller (35–40 mm^3^) tumors appeared to drastically reduce lung metastases. Alternatively, it is possible that anti-tumor immune responses were elicited by exposure of tumor antigens after destruction of 4T1 cells within the primary tumor by the OncSyn and OncdSyn viruses. In this regard, a fusogenic oncolytic HSV-1 Synco-2D was reported to elicit anti-tumor immune responses when studied in a similar animal model of mammary carcinoma utilizing 4T1 cells [14]. A strong T-cell response was reported also by an HSV-2 derivative oncolytic virus FusOn-H2 effectively treating primary and metastatic mammary tumors *in vivo *[15].

## Conclusion

Overall, our results showed that both OncSyn and OncdSyn viruses can efficiently reduce the primary and metastatic growth of 4T1 tumors established in immunocompetent mice. It is expected that these viruses would be even more efficacious against human breast cancer tumors by virtue of the fact that they can replicate substantially more efficiently (more than one log) in human than mouse cells. The availability of both OncSyn and OncdSyn viruses as bacterial artificial chromosomes will enable the generation of additional recombinant viruses that carry multiple anti-tumor and immunomodulatory transgenes, which could further enhance the anti-tumor efficacy of these viruses.

## Materials and methods

### Cells

African green monkey kidney (Vero) cells and mouse mammary tumor cells (4T1) [40] were obtained from the American Type Culture Collection (Manassas, VA). Vero cells were maintained in Dulbecco's modified Eagle's medium (Gibco-BRL; Grand Island, N.Y.), supplemented with 10% fetal calf serum (FCS) and antibiotics. 4T1 cells were maintained in RPMI 1640 medium (Hyclone, Logan, UT) containing 10% FCS. The cultures were maintained at 37°C in a humidified atmosphere of 5% CO_2_/95% air.

### Construction of the doubly fusogenic recombinant virus HSV-1 OncdSyn

The previously published OncSyn viral genome recovered as a bacterial artificial chromosome (bac) into *E. coli *(pOncSyn) [37] was used for the construction of pOncdSyn bac plasmid utilizing a new methodology – the double-red mutagenesis technique in *E. coli *[39] enabling the markerless introduction of the gKsyn1 mutation (Ala-toVal at aa 40). The OncdSyn virus was recovered after transfection of Vero cells with the pOncdSyn plasmid. The OncdSyn viral genome and the pOncdSyn bac were extensively characterized by diagnostic PCR and DNA sequencing to ensure the stability of the viral genomes, the presence of the parental Onc deletions and the presence of the gKsyn1 mutation within the gK gene, as described previously for the OncSyn virus [37].

### Phenotypic characterization and replication kinetics of the OncSyn and OncdSyn viruses

Cells (both Vero and 4T1) were seeded into 6-well plates and infected the following day (when they reached approximately 95% confluency) with the OncSyn or OncdSyn viruses at a multiplicity of infection (MOI) ranging from 0.001–1 plaque forming units per cell (PFU/cell). Cells were cultured in a maintenance medium (containing 2% FCS) and were left for 2 days to allow for the plaques and the cell fusion to develop. Photographs of the infected cells were taken using a fluorescence microscope. For assessment of viral plaque morphologies, Vero and 4T1 cells were infected with HSV-1(F), OncSyn or OncdSyn viruses and visualized after immunohistochemistry at 48 hours post-infection (h.p.i.) using horseradish peroxidase-conjugated anti-HSV antibody (Dako, Carpinteri, CA) and Novared substrate development kit (VectorLabs, Burlingame, CA).

To determine the replication kinetics of the viruses, one-step growth kinetics were performed as described previously [48,49]. Briefly, nearly confluent monolayers of either Vero or 4T1 cells were infected with each virus at an MOI of 2 at 4°C for 1 h. Thereafter, virus was allowed to penetrate for 2 h at 37°C. Any remaining extracellular virus was inactivated by low-pH treatment with phosphate buffered saline at pH 3.0. Cells and supernatants were harvested immediately thereafter (0 h) or after 12 or 24 h of incubation at 37°C. Virus titers were determined by endpoint titration of virus stocks on Vero cells.

### Animal experiments

Female Balb/c mice were obtained from Harlan (Indianapolis, IN) and housed in an animal room which was kept at 25°C with a 12 hour light-dark cycle. All experimental procedures involving animals were approved by the institutional animal care and use committee (IACUC) of the Louisiana State University. At 6–7 weeks of age the animals (19–20 g body weight) were implanted subcutaneously in the interscapular area with 1 × 10^5^viable 4T1 cells suspended in 0.2 ml of PBS using a 27 gauge needle. Body weights were determined weekly, and tumor sizes were monitored beginning 7 days after tumor inoculation by direct measuring with a digital microcaliper. Tumor volumes were calculated using the following formula: volume = (length × width × height)/2. At an average tumor volume of approximately 80–90 mm^3 ^(first experiment) or 35–40 mm^3 ^(second experiment), animals were randomized into 3 groups (first experiment) or 2 groups (second experiment) using a randomization plan. The groups of mice received 3 intratumoral injections of the OncSyn, OncdSyn viral particles, or PBS every four days for the first experiment and injections of the OncdSyn or PBS every third day for the second experiment. Each tumor was injected with approximately 1 × 10^6 ^viruses per injection in 250 μl volume, while control mice received 250 μl of PBS. Injections were performed slowly at 3 different sites per tumor. On day 42 for the first experiment and day 33 for the second experiment after initial tumor cell implantation, mice were humanely euthanized in a CO_2 _chamber and subjected to gross as well as microscopic histological examination. Lung and other internal organ metastases were counted using a dissecting microscope after placing the resected organs in fixative for 24 hours. The primary tumor site, lungs, heart, liver, spleen, and kidneys from each animal were fixed in 10% neutral buffered formalin, trimmed, paraffin embedded, sectioned, stained with hematoxylin and eosin (H&E), and evaluated by light microscopy.

### Statistical methods and analyses

The SAS^® ^statistical package (Version 9.1.3) was used for the analyses of the *in vivo *studies. Distributions were examined for normality using the UNIVARIATE procedure with a Shapiro-Wilk test of normality. For the repeated measures part of the analyses of tumor volumes and tumor weights, the GLM procedure was used to conduct a repeated measures design analyzed as a split-plot arrangement of treatments with TREATMENT (OncSyn, OncdSyn, and PBS) and MOUSE within TREATMENT as main plot factors. Subplot factors included PERIOD (days of measurements) and TREATMENT by PERIOD interaction. When overall analyses determined significance (p = 0.05), Tukey's HSD test was used to examine pairwise differences for main effects, and pairwise comparisons of least square means with regard to interaction effects were examined with preplanned t-tests. The Wilcoxon Two-Sample test was used to analyze the difference of lung metastatic node counts between PBS and OncdSyn groups.

## Competing interests

The authors declare that they have no competing interests.

## Authors' contributions

AI performed most of the experiments and participated in drafting the manuscript, VNC participated in the construction and characterization of the viruses, AB was involved in the design and conduction of in vivo studies, ATD participated in pathological analysis and interpretation of data, MTK performed the statistical analyses, KGK was overall responsible for the project and for the preparation of the manuscript.
